# epiPATH: an information system for the storage and management of molecular epidemiology data from infectious pathogens

**DOI:** 10.1186/1471-2334-7-32

**Published:** 2007-04-20

**Authors:** Alicia Amadoz, Fernando González-Candelas

**Affiliations:** 1 Institut Cavanilles de Biodiversitat i Biologia Evolutiva and Departament de Genètica, Universitat de València, Spain

## Abstract

**Background:**

Most research scientists working in the fields of molecular epidemiology, population and evolutionary genetics are confronted with the management of large volumes of data. Moreover, the data used in studies of infectious diseases are complex and usually derive from different institutions such as hospitals or laboratories. Since no public database scheme incorporating clinical and epidemiological information about patients and molecular information about pathogens is currently available, we have developed an information system, composed by a main database and a web-based interface, which integrates both types of data and satisfies requirements of good organization, simple accessibility, data security and multi-user support.

**Results:**

From the moment a patient arrives to a hospital or health centre until the processing and analysis of molecular sequences obtained from infectious pathogens in the laboratory, lots of information is collected from different sources. We have divided the most relevant data into 12 conceptual modules around which we have organized the database schema. Our schema is very complete and it covers many aspects of sample sources, samples, laboratory processes, molecular sequences, phylogenetics results, clinical tests and results, clinical information, treatments, pathogens, transmissions, outbreaks and bibliographic information. Communication between end-users and the selected Relational Database Management System (RDMS) is carried out by default through a command-line window or through a user-friendly, web-based interface which provides access and management tools for the data.

**Conclusion:**

epiPATH is an information system for managing clinical and molecular information from infectious diseases. It facilitates daily work related to infectious pathogens and sequences obtained from them. This software is intended for local installation in order to safeguard private data and provides advanced SQL-users the flexibility to adapt it to their needs.

The database schema, tool scripts and web-based interface are free software but data stored in our database server are not publicly available. epiPATH is distributed under the terms of GNU General Public License. More details about epiPATH can be found at .

## Background

Infectious disease is defined as any illness caused by a specific pathogenic microorganism or its toxic product that results from the transmission of that agent or its product from an infected to a susceptible host, either directly or indirectly through an intermediate [1]. There are many different infectious pathogens that cause this kind of diseases and also some of them can be present as multiple infections in the same individual, such as patients coinfected with Hepatitis C Virus (HCV) and Human Immunodeficiency Virus (HIV).

Molecular genetic information is being increasingly used in the epidemiological study of infectious diseases to understand transmission, virulence or resistance patterns of microorganisms [2], pathogen evolution and host-pathogen interactions [3,4], pathogen resistance to treatments, susceptibility to disease, etc. In the study of pathogenic processes different kinds of demographical, epidemiological, clinical and molecular data are important to obtain a complete view and a more comprehensive understanding of the infectious disease. In most cases, this kind of studies are developed by different institutions, sometimes geographically disperse, collaborating with each other by contributing of different types of information.

To study the interaction between a patient and a pathogen from a population, evolutionary and epidemiological perspective, a huge amount of molecular sequences is needed and current molecular techniques provide, with increasing affordability, this kind of data. An essential component to perform this kind of studies is having all these data properly organized and accessible [5,6] to all implicated researchers. In the case that a study is under development and unfinished, users may not want to have their own information being publicly accessible. These points, in addition to a multi-user support capable system, are central aspects that must be taken into account when choosing a software to perform these tasks.

Until now, programs to perform molecular epidemiology studies of infectious diseases were, on one hand, devoted to the storage of information on patients and, on the other, public databases repositories [7] or internal flat files to store and retrieve sequences of pathogens. Currently, technologies to develop an information system are available as free software and researchers are able to build their own data management system. But this is a time-consuming, costly process which requires considerable expertise. Consequently and since there is no public database schema that incorporates clinical and epidemiological information about patients and molecular information about pathogens, we have developed a very complete and flexible information system, composed by a main database and a web-based interface, that integrates both types of data and satisfies requirements of good organization, simple accessibility, data security and multi-user support system.

## Implementation

An information system consists of a group of elements related according to some rules, which provides the necessary information to fulfil its purposes. The goals of such an information system are the collection, processing, storage, production and presentation of data. Our information system is composed by a main MySQL [8] database and a web-based interface.

### Database design

**Figure 1:**
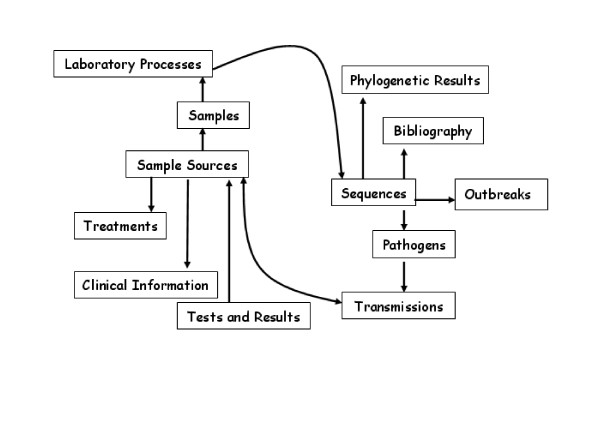
Flow diagram of information collected in the information system.[20]

From the moment a patient arrives to a hospital or health centre to the analysis of sequences of the infectious pathogen in the laboratory, lots of information are collected. We have extensively discussed with researchers and physicians that actually work in these and related fields about their needs and finally we have divided all the sources of information into 12 conceptual modules around which the database schema has been organized (Table 1). Figure 1 shows the flow chart of information involved along this process. The flow diagram of information starts with the SAMPLE SOURCES module which refers to those places where a pathogen can be found, either in patients or the environment. Then, we can obtain CLINICAL INFORMATION of a patient when (s)he goes to physician, who asks the patient to get some CLINICAL TESTS whose RESULTS correspond to the patient. Moreover, we can have information about the type of TRANSMISSION with respect to how or by whom was the patient infected and who (s)he may have infected to. Then, the physician gives a TREATMENT to the patient and samples of the pathogen can be collected. Samples are processed at the LABORATORY where SEQUENCES are obtained. These sequences correspond to a specific PATHOGEN which is associated to a type of transmission and, in some cases, to an OUTBREAK. Furthermore, sequences can be analyzed with PHYLOGENETIC METHODS and eventually be PUBLISHED in different studies.

**Table 1:** Conceptual modules of the database schema.

**Module**	**Information**
Bibliography	Bibliographic references, authors, researchers and research groups
Clinical information	Diseases, symptoms, signs, risk factors, protector factors, clinical information, pathogen's tests, vaccines and vaccinations
Laboratory processes	Laboratory processes, extractions, regions, primers, amplifications, sequencing and typing
Outbreaks	Outbreaks
Pathogens	Pathogens
Phylogenetic results	Alignments and phylogenetic trees
Sample sources	Patients, environments, hospitals/health centers and patients' information
Samples	Samples and storage
Sequences	Sequences
Test and Results	Tests^1^, laboratories, test results and results
Transmissions	Transmissions
Treatments	Treatment names and dates

^1 ^Tests from CLIA [20] and some added from our laboratory.

**Figure 2:**
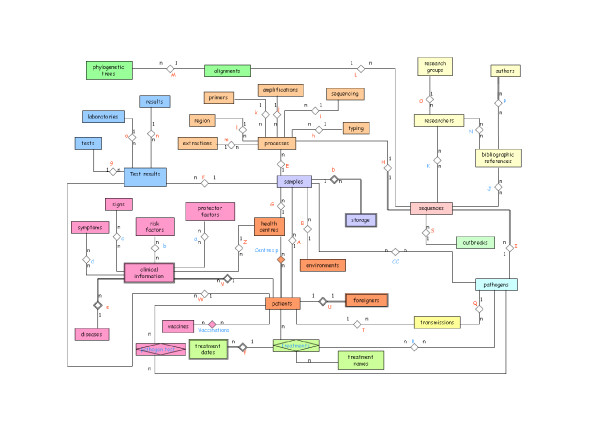
**Entity-Relationship schema of the epiPATH molecular epidemiology database**. A named square shows an entity, lines and rhombus show the relationships and valid requests are represented by 1-1 (one to one), 1-n (one to many) and n-n (many to many). Entities are grouped by different colours showing the 12 conceptual modules.

**Figure 3:**
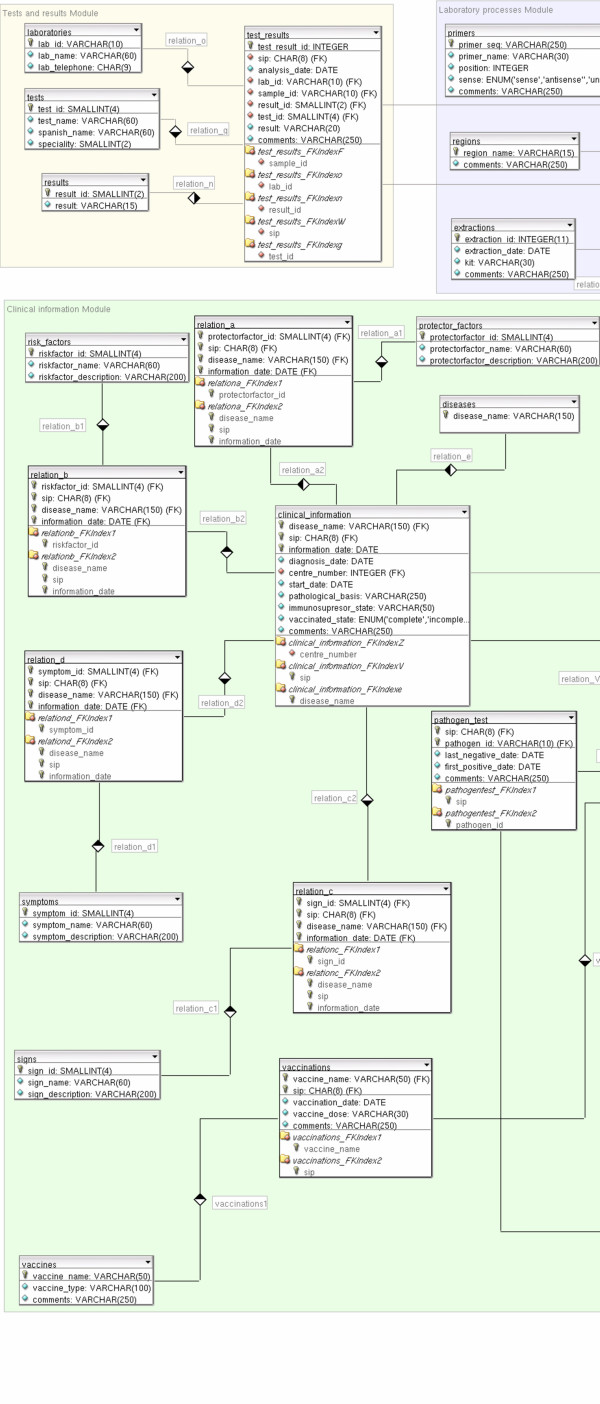
**Entity-Relationship diagram developed with DBDesigner 4**. Database schema with detailed tables and relationships. Conceptual modules are emphasized in different colours as background rectangles. To preserve visibility, the figure has been divided into three parts (Figures 3–5, from left to right).

**Figure 4:**
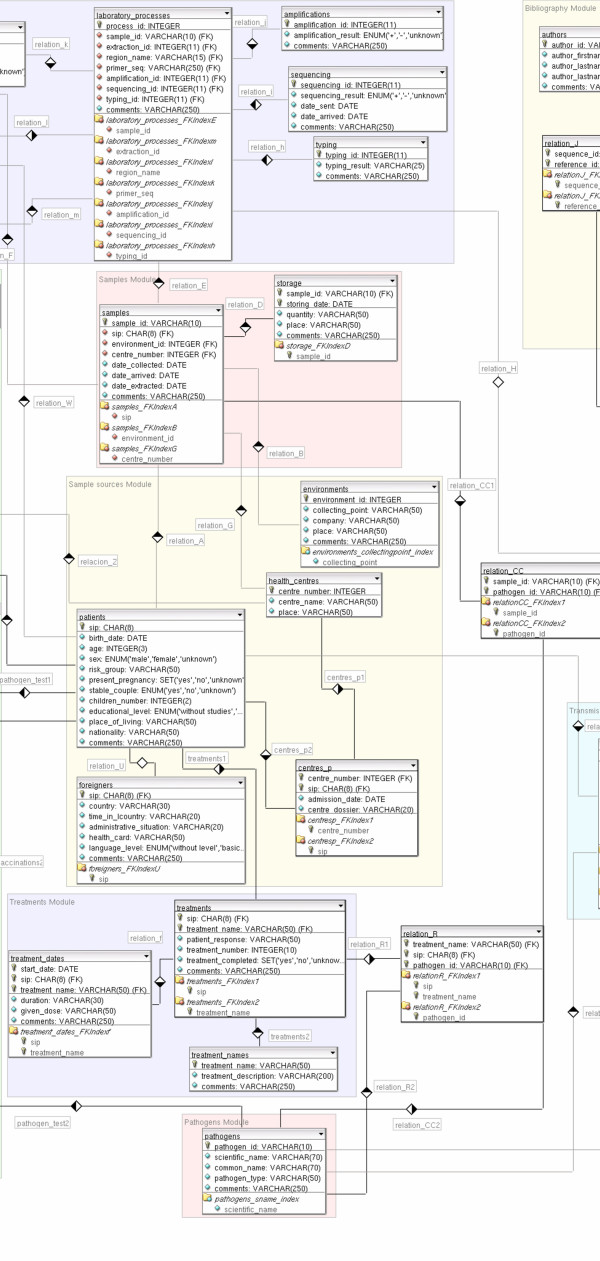
**Entity-Relationship diagram developed with DBDesigner 4**. Database schema with detailed tables and relationships. Conceptual modules are emphasized in different colours as background rectangles. To preserve visibility, the figure has been divided into three parts (Figures 3–5, from left to right), from left to right).

**Figure 5:**
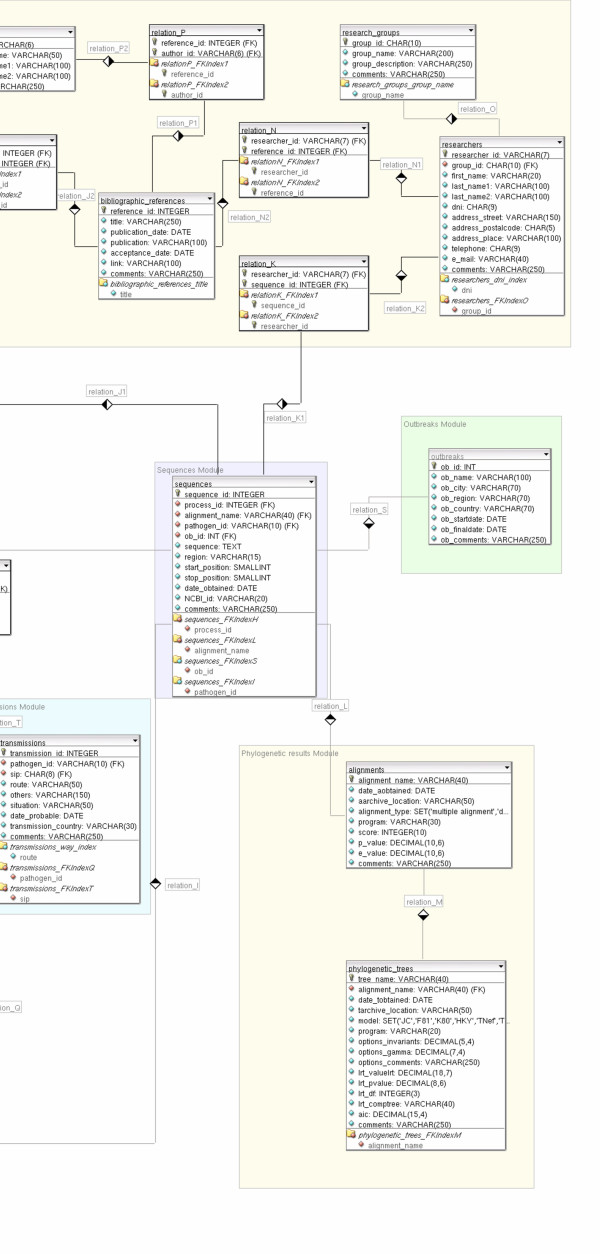
**Entity-Relationship diagram developed with DBDesigner 4**. Database schema with detailed tables and relationships. Conceptual modules are emphasized in different colours as background rectangles. To preserve visibility, the figure has been divided into three parts (Figures 3–5, from left to right).

The data model chosen to represent data is the relational model [9], composed by relations, that basically are tables in which a row is called *tuple *and a column is called *attribute*. An Entity-Relationship (ER) diagram defines the entities of the database and their relationships, so we can have a global view of the information stored, as shown in Figure 2. In this diagram the structures that set up the information system with the restrictions that limit valid requests are represented. Entities and attributes are extended in a diagram developed with DBDesigner 4 [10] program (Figures 3, 4, 5). A detailed description of each field of the relational database tables is provided as Additional file 1.

In designing the database we have taken into account all the existing infectious pathogens, either virus or bacteria, so the system can store information from different organisms at the same time. This is very useful mainly in cases of multiple infections, when studies involve more than one pathogen per patient and all relevant information (clinical, epidemiological and molecular) should be available. In these situations, it is easier for users and system administrators to deposit and control all the information in the same database schema instead of using different or independent database schemas. Moreover, the infectious pathogen general design contributes to the versatility and suitability of this software that can be used in many different ways as needed by different users. For example, epiPATH could be used for a unique pathogen exclusively or multiple pathogens simultaneously related studies: a user may start studying one single pathogen, such as HCV, and later he starts a new project with HCV/HIV coinfected patients or with a completely new pathogen. In this case there is no need to modify this software and, obviously, the learning curve is reduced to a minimum. Furthermore, although this database schema has been designed for multiple pathogens, it can be used as a database for individual pathogens on a per user basis by means of the *Views *utility of MySQL. A *view *is a virtual or logical table composed for the result of a query. Using *views *allows different accesses to the same database schema, either for different users, pathogens or any database characteristic of interest. *Views *can be created as needed by the system administrator and they also contribute to the security and integrity of the data stored preventing a user from editing or deleting other user's data.

Conceptual modules, in which information is divided, add versatility to this software so that users choose which kind of information is relevant for their studies by storing that part of the database schema. Moreover, advanced SQL-users could adapt it to their needs since epiPATH is distributed under GNU license.

### Database architecture

The molecular epidemiology database consists of 50 tables and was built using the MySQL RDMS running on a Linux Fedora system. We used *InnoDB *tables because they allow transactions. Currently, it is implemented in MySQL Server version 5 but it also has been implemented in earlier versions of MySQL Server without any problem. Database design and scripts to create the schema have been developed and obtained with DBDesigner 4. Scripts were debugged and implemented into the RDMS with MySQL Query Browser [11]. These tools have been selected because of their free availability and wide use, as well as their reliability and flexibility to run under different environments.

### Web-based interface design

**Figure 6:**
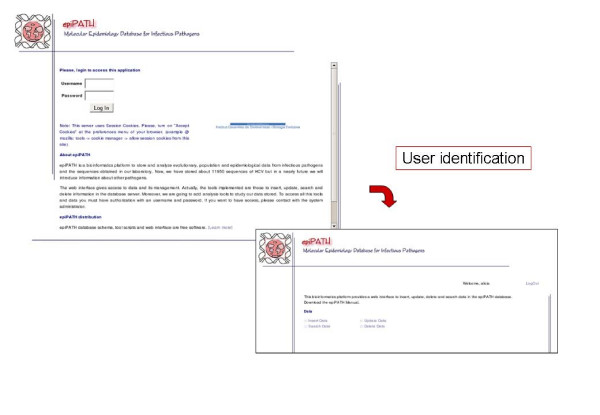
Screenshots of epiPATH main pages.

**Figure 7:**
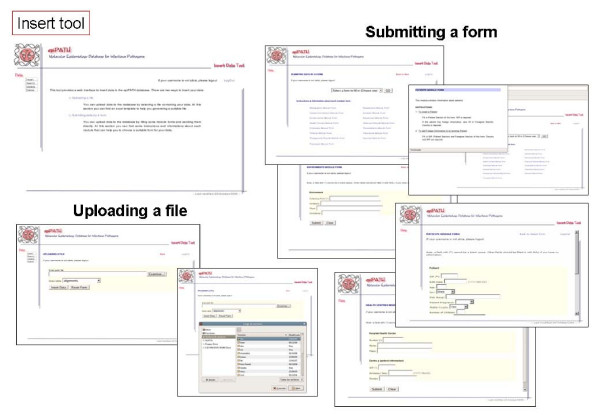
Screenshots of epiPATH insert tool.

**Figure 8:**
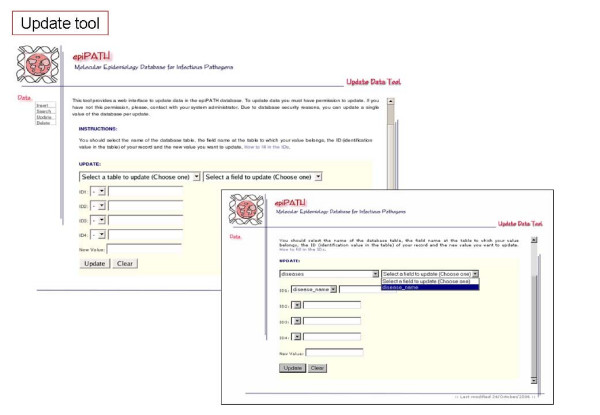
Screenshots of epiPATH update tool.

**Figure 9:**
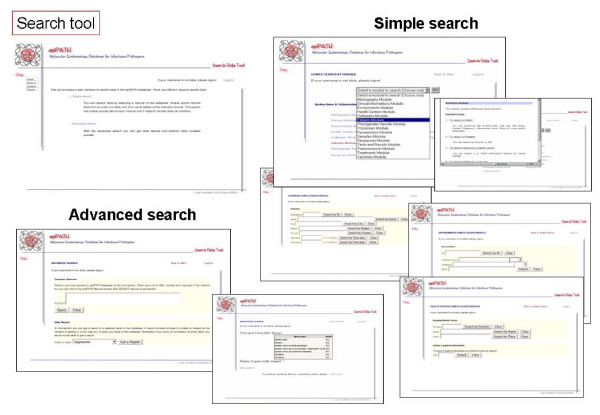
Screenshots of epiPATH search tool.

**Figure 10:**
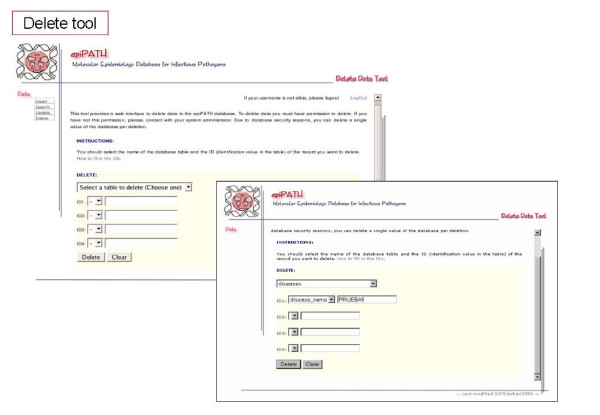
Screenshots of epiPATH delete tool.

Communication between users and the selected RDMS is carried out by default through a command-line interface. However, this is not an easy way for users with poor computer skills, and we have developed a web-based interface providing access to the data and its management (Figures 6, 7, 8, 9, 10). This user-friendly interface has a simple usage mainly through diverse forms of the implemented tools. Currently, the tools implemented are those to insert (Figure 7), update (Figure 8), search (Figure 9) and delete (Figure 10) information in the database server. Each user needs a username and a password to access all the web interface tools and the information system. In fact, the username and password needed to access the web-based interface are the same used for accessing the MySQL RDMS through a command-line shell. This procedure improves system security by relying on passwords on the RDMS instead of on an external system file.

There are two ways to introduce data into the database: users can upload a file containing all data to be inserted or they may introduce data by filling a specific web form. The former method is indicated when the user wants to add many records into a database table and the later when the number of records to insert is reasonably low. There is a specific web form for nearly every conceptual module of the database schema. Searches can be performed by specific web forms, as insertion tools, by entering a complex query, that allows a wide range of options to construct an appropriate query for the user, or obtaining reports of a single table from the database. Reports make possible to retrieve information already stored in each database table without knowing exactly what to look for. Updating and deleting data can be done only on one record at a time due to security reasons. When using the web-based interface, all data changes such as insertions, deletions and updates are recorded in another database that can only be accessed by the system administrator. This feature improves the security of our information system and it is also distributed with this software.

When working in molecular epidemiology, the information needed to perform some studies can originate from different sources, hospitals/health centres and laboratories. As a consequence, some inconsistencies in the data might appear, mostly related to the limits and genotype of sequences. To overcome this problem, we have developed and installed some scripts in the web-based interface that warn users about the existence of incoherent data.

### Web-based interface architecture

The web interface has been developed in HTML language and includes scripts in Javascript [12] and PHP [13], both platform independent languages. It is accessible through an HTTP Apache server [14].

### Data security

A first level of security implemented in this software is the identification of the user to log in. Both through the command-line shell or the web-based interface, the user must be identified with a username and a password, both corresponding to those implemented in the local MySQL server. A second level of security is provided by the possibility of using *Views *to allow different users to access and manage different parts of the database, either by pathogen, project, or any other selective criterion. We distribute this software both with and without this utility implemented in the web-based interface and the database schema. With the *Views *utility it is possible to create more levels of virtual tables and therefore to increase the security level as desired. A third level is a log out script implemented on the web-based interface that alerts users after 25 minutes of inactivity and automatically ends the current session 5 minutes later if inactivity continues. A fourth level is a secondary database schema exclusively accessed by the system administrator that stores information about whom, where and when an insertion, deletion or update through the web-based interface was performed. A final security level for the data is a backup of the entire database that must be performed by the local system administrator.

## Results and discussion

There is a need for an information system to manage data obtained from different molecular epidemiology studies related to infectious pathogens. We aim to overcome this lack in an efficient manner with our database schema and tools software. epiPATH is an information system for managing data about clinical and molecular information of infectious diseases, which is available for download and local installation, in order to help researchers in related fields in their daily tasks in a laboratory and collaborative projects with other institutions, preserving the information from public access given the sensitivity of the data under analysis. We provide both the database schema and web-based interface, with and without *views *utility implemented that are available from the epiPATH website. Additionally, a complete manual for users and administrators and a detailed documentation of the database schema can be found at the same site.

### Testing

Once the information system was implemented, we performed some tests with data from the Hepatitis C Virus and its disease. Sequences and clinical data derived from previous studies in our research group [15][16]. Tests included 73 samples, 11952 viral sequences and clinical information of 88 patients.

### Usability

epiPATH is designed to be a local software for laboratories and institutions interested in molecular epidemiology, population genetics and evolutionary biology of infectious diseases. The database schema covers all relevant information of these fields and becomes a very complete and versatile tool. Due to its modular conceptual tables it can be used in a full manner or only in those parts of information that a researcher is actually interested in. Being a multi-pathogen designed database, it can be used as virtual database for a single pathogen or on a per user basis by means of the *Views *utility. At the same time, it incorporates all data in a unique schema for those cases when multiple infections and comparisons between different pathogens are relevant, and additionally, being a single software for many different users and projects contributes to the internal daily work and data organization of many different institutions. The web-based interface is a very easy-to-use tool that allows the management of users' data stored in a local MySQL database server. The database schema and the web-based interface of epiPATH are open source software under the GNU license and can be modified by end users as needed.

We performed a usability test  [17][18][19] with 6 participants to determine whether the design and tools implemented allow users to manage information easily through the interface and to gather information from final users to enhance our current version of the software. The study was performed with researchers from a molecular biology lab (Table 2) with a very similar profile to that of the final users of this platform.

**Table 2:** Summary of relevant features of the participants in the usability test of epiPATH.

Feature	Value
Number of participants	6
Average age	27.33 years
Academic studies	100% PhD students in Biology
Gender	50% Male, 50% Female
Previous experience with databases	83.33% Yes, 16.66% No
Previous experience with SQL language	100% No
Previous experience with epiPATH	100% No
Previous experience in navigation through web sites	100% Yes

Subjects were given a 10-minute tutorial and they were asked to complete 5 different tasks on a test database. Each participant was tested and recorded independently. The main results are summarized in Table 3. All the tasks were graded from "very easy" to "easy" on a five-level scale. The only errors were made during the "Search" task, a reflection of the initial difficulties of queries in the SQL language, but, on the other hand, users also appreciated the simplicity of forms and reports. We also received some suggestions for improvement in future updates of the platform.

**Table 3:** Summary of results in the usability test. Five tasks were considered: (1) entering into epiPATH interface, (2) adding data into epiPATH database through our interface, (3) searching data through epiPATH interface, (4) updating data through epiPATH interface, and (5) deleting data from epiPATH database.

**Task**	**Average time in sec (range)**	**Average number of errors**	**Average difficulty grade^1^**
1	34 (8–72)	0	1
2	113.5 (88–145)	0	1
3	93 (65–124)	0.5	1.5
4	46.8 (35–63)	0	1.33
5	30.8 (5–124)	0	1.5

^1^Difficulty grade: from 1 (very easy) to 5 (very difficult).

### Future directions

In the near future we plan to develop and integrate in the web-based interface new and already existing analytical tools for this kind of data in order to study thoroughly infectious pathogens from molecular, population, epidemiology and evolutionary points of view.

## Conclusion

epiPATH is an open source information system for storing and managing data from clinical and molecular information of infectious diseases, which is available for download and local installation, in order to help researchers in related fields in their daily tasks in the laboratory and in collaborative projects with other institutions, preserving the information from public access given the sensitivity of the data under analysis. It is a very complete, suitable and versatile tool and an unique software for many different users and projects that satisfies requirements of good organization, simple accessibility, data security and multi-user support system.

## Availability and requirements

**Project name: **epiPATH

**Project home page: **

**Operating system(s): **Client: a web broswer (Firefox, Mozilla, Explorer...); Server: a MySQL Server (epiPATH distribution with views requires MySQL 5 or higher) running on UNIX/Linux or Microsoft Windows systems.

**Programming language: **Web-based interface: HTML, PHP and JavaScript; Database: SQL/MySQL.

**Other requirements: **Apache HTTP Server, MySQL client and MySQL Query Browser.

**License: **GNU General Public License.

The epiPATH software is freely available from the above web site. Data stored in our database server are not publicly available.

## Abbreviations

RDMS – relational database management system

SQL – structured query language

HCV – hepatitis C virus

HIV – human immunodeficiency virus

ER – entity-relationship

HTML – hypertext markup language

PHP – php hypertext pre-processor

HTTP – hypertext transfer protocol

## Authors' contributions

AA developed and implemented the database schema and the interface. FG designed the project and supervised its development. AA and FG wrote the manuscript.

## Pre-publication history

The pre-publication history for this paper can be accessed here:



## Supplementary Material

Additional file 1 Description of database terms. Detailed information of each field and table of the database schema. Click here for file
