# Supplementary material for: epiPATH: an information system for the storage and management of molecular epidemiology data from infectious pathogens
Source: BMC Infect Dis. 2007 Apr 20;7:32. doi: 10.1186/1471-2334-7-32 (PMC1868736; doi:10.1186/1471-2334-7-32)
Supplement: Additional file 1 — Description of database terms. Detailed information of each field and table of the database schema. [file 1471-2334-7-32-S1.doc]

**Supplementary data**

**1. Description of database terms**

**Important note:** Fields that are mandatory are defined in the database table as NOT NULL. This means that this field cannot be a blank field. If you have no information in a field of this type, it is recommended to fill in it with **NULL**.

**Sequences Module:**

*Sequences* table:

- **sequence_id**: unique sequence identifier (by default a unique number between 0 and 4294967295). It is a mandatory field.
- **process_id**: unique laboratory process identifier to which the above sequence belongs (links to *laboratory_processes* table). It is a mandatory field.
- **alignment_name**: unique alignment identifier in which the above sequence appears (links to *alignments* table).
- **pathogen_id**: unique pathogen identifier to which the above sequence belongs (links to *pathogens* table). It is a mandatory field.
- **ob_id**: unique outbreak identifier to which the above sequence belongs (links to *outbreaks* table).
- **sequence**: complete sequence obtained from the analyzed genome region (ATCG letter code, maximum length 65535 characters). It is a mandatory field.
- **region**: where the sequence above belongs (region name, maximum 15 characters).
- **start_position**: where the sequence above starts (initial nucleotide number between 0 and 65535). It is a mandatory field.
- **stop_position**: where the sequence above ends (final nucleotide number between 0 and 65535). It is a mandatory field.
- **date_obtained**: date when the sequence was obtained (date format: YYYY-MM-DD).
- **NCBI_id**: sequence identification number at NCBI databases (maximum 20 characters).
- **comments**: sequence information not stored before in any field of this table (maximum 250 characters).

**Outbreaks Module:**

*Outbreaks* table:

- **ob_id**: unique outbreak identifier (by default a unique number between 0 and 4294967295). It is a mandatory field.
- **ob_name**: name of the outbreak (maximum 100 characters). It is a mandatory field.
- **ob_city**: name of the city where the outbreak is localized (maximum 70 characters).
- **ob_region**: name of the region where the outbreak is localized (maximum 70 characters).
- **ob_country**: name of the country where the outbreak is localized (maximum 70 characters).
- **ob_startdate**: date when the outbreak started (date format: YYYY-MM-DD).
- **ob_finaldate**: date when the outbreak ended (date format: YYYY-MM-DD).
- **ob_comments**: outbreak information not stored before in any field of this table (maximum 250 characters).

**Samples Module:**

*Samples* table:

- **sample_id**: unique sample identifier used at the laboratory (numbers and/or letters code, maximum 10 characters). It is a mandatory field.
- **SIP**: unique numerical personal identifier of each patient from which a sample comes (links to *patients* table).
- **environment_id**: unique environment identifier from which a sample comes (links to *environments* table).
- **centre_number**: unique hospital or health centre identifier from where a sample comes (links to *health centres* table).
- **date_collected**: date when the sample was taken from the patient (date format: YYYY-MM-DD).
- **date_arrived**: date when the sample arrived to the laboratory (date format: YYYY-MM-DD).
- **date_extracted**: date when the sample was extracted in the laboratory (date format: YYYY-MM-DD).
- **comments**: sample information not stored before in any field of this table (maximum 250 characters).

*Storage* table:

- **sample_id**: unique sample identifier used at the laboratory (numbers and/or letters code, maximum 10 characters). It is a mandatory field.
- **storing_date**: date when a sample was stored in the laboratory (date format: YYYY-MM-DD). It is a mandatory field.
- **quantity**: number of tubes, plates, ml… of a sample stored at the date above (maximum 50 characters).
- **place**: where the sample above is stored (maximum 50 characters).
- **comments**: storage information not stored before in any field of this table (maximum 250 characters).

**Sample Sources Module:**

*Patients* table:

- **SIP**: unique numerical personal identifier of each patient (8 characters). It is a mandatory field.
- **birth_date**: date of birth of a patient (date format: YYYY-MM-DD).
- **age**: in years (number of 3 digits).
- **sex**: (select one from this options: ‘male’, ‘female’, ‘unknown’). It cannot be a blank space.
- **risk_group**: risk group to which a patient belongs (maximum 50 characters).
- **present_pregnancy**: it refers to a patient if she is pregnant when she goes to the hospital or health centre (select one from this options: ‘yes’, ‘no’, ‘unknown’). It can be a blank space.
- **stable_couple**: information about stability situation of a patient (select one from this options: ‘yes’, ‘no’, ‘unknown’). It cannot be a blank space.
- **children_number**: number of children of a patient (number between 0 and 99).
- **educational_level**: information about the patient’s studies (select one from these options: ‘without studies’, ‘primary school’, ‘secondary school’, ‘university’, ‘doctor’, ‘unknown’). It cannot be a blank space.
- **place_of_living**: place where a patient lives (maximum 50 characters).
- **nationality**: patient’s nationality (maximum 50 characters).
- **comments**: patient information not stored before in any field of this table (maximum 250 characters).

*Foreigners* table:

- **SIP**: unique numerical personal identifier of each patient (links to *patients* table). It is a mandatory field.
- **country**: country of origin to which the patient belongs (maximum 30 characters). It is a mandatory field.
- **time_in_lcountry**: time since arrival to local country (maximum 20 characters).
- **administrative_situation**: administrative situation in which a patient is (maximum 20 characters).
- **health_card**: if a patient has a health card or is obtaining it (maximum 50 characters).
- **language_level**: local language level of a patient (select one of this options: ‘without level’, ‘basic level’, ‘advanced level’, ‘perfect’, ‘unknown’). It cannot be a blank space.
- **comments**: foreigner information not stored before in any field of this table (maximum 250 characters).

*Health centres* table:

- **centre_number**: unique hospital or health centre identifier (between 0 and 4294967295). It is a mandatory field.
- **centre_name**: name of the hospital or health centre from where a patient comes (maximum 50 characters).
- **place**: place at where the hospital or health centre is located (maximum 50 characters).

*Centres p* table:

- **centre_number**: unique hospital or health centre identifier (between 0 and 4294967295). It is a mandatory field.
- **SIP**: unique numerical personal identifier of each patient (links to *patients* table). It is a mandatory field.
- **admission_date**: date of patient admission at the hospital or health centre (date format: YYYY-MM-DD).
- **centre_dossier**: dossier number of a patient at the hospital or health centre that the patient comes from (maximum 20 characters).

*Environments* table:

- **environment_id**: unique environment identifier (by default a unique number between 0 and 4294967295). It is a mandatory field.
- **collecting_point**: place where a sample was collected (maximum 50 characters). It is a mandatory field.
- **company**: company where the collecting point is located (maximum 50 characters).
- **place**: place where the company or collecting point is located (maximum 50 characters).
- **comments**: environment information not stored before in any field of this table (maximum 250 characters).

**Pathogens Module:**

*Pathogens* table:

- **pathogen_id**: unique pathogen identifier (maximum 10 characters). It is a mandatory field.
- **scientific_name**: pathogen scientific name (maximum 70 characters). There cannot be two identical scientific names.
- **common_name**: pathogen common name (maximum 70 characters).
- **pathogen_type**: information about the characteristics of the pathogen (maximum 50 characters).
- **comments**: pathogen information not stored before in any field of this table (maximum 250 characters).

**Transmissions Module:**

*Transmissions* table:

- **transmission_id**: unique transmission identifier (by default a number between 0 and 4294967295). It is a mandatory field.
- **pathogen_id**: unique pathogen identifier (links to *pathogens* table). It is a mandatory field.
- **SIP**: unique numerical personal identifier of each patient (links to *patients* table). It is a mandatory field.
- **route**: way of transmission (most likely) of a pathogen (maximum 50 characters). It is a mandatory field.
- **others**: other routes of transmission (maximum 150 characters).
- **situation**: at which situation a transmission was produced (maximum 50 characters).
- **date_probable**: when the transmission was produced (date format: YYYY-MM-DD).
- **transmission_country**: country in which the transmission was produced (maximum 30 characters).
- **comments**: transmission information not stored before in any field of this table (maximum 250 characters).

**Treatments Module:**

*Treatments* table:

- **SIP**: unique numerical personal identifier of each patient (links to *patients* table). It is a mandatory field.
- **treatment_name**: unique treatment name identifier (links to *treatment names* table). It is a mandatory field.
- **patient_response**: response of the patient to the treatment above (maximum 50 characters).
- **treatment_number**: if the treatment administered to the patient is the first or other number of treatment (maximum 10 digits).
- **treatment_completed**: information on whether the patient has finished the above treatment (select one option between: ‘yes’, ‘no’, ‘unknown’). It can be a blank space.
- **comments**: treatment information not stored before in any field of this table (maximum 250 characters).

*Treatment names* table:

- **treatment_name**: unique treatment name identifier (maximum 50 characters). It is a mandatory field.
- **treatment_description**: characteristics and description of the treatment (maximum 200 characters).
- **comments**: treatment name information not stored before in any field of this table (maximum 250 characters).

*Treatment dates* table:

- **start_date**: when the treatment started (date format: YYYY-MM-DD). It is a mandatory field.
- **SIP**: unique numerical personal identifier of each patient (links to *patients* table). It is a mandatory field.
- **treatment_name**: unique treatment name identifier (links to *treatment names* table). It is a mandatory field.
- **duration**: time that the treatment lasts or how often it is administered to the patient (maximum 30 characters).
- **given_dose**: dose administered to the patient, drug and dose included (maximum 50 characters).
- **comments**: treatment date information not stored before in any field of this table (maximum 250 characters).

**Tests and Results Module:**

*Test results* table:

- **test_result_id**: unique test result identifier of a sample, a patient or both (by default a number between 0 and 4294967295). It is a mandatory field.
- **SIP**: unique numerical personal identifier of each patient (links to *patients* table).
- **analysis_date**: when the analysis test was done (date format: YYYY-MM-DD).
- **lab_id**: unique laboratory identifier where the test was done (links to *laboratories* table).
- **sample_id**: unique sample identifier analyzed (links to *samples* table).
- **result_id**: unique result identifier of the test (links to *results* table).
- **test_id**: unique test identifier to which result corresponds (links to *tests* table). It is a mandatory field.
- **result**: numeric result value or qualitative result (maximum 20 characters).
- **comments**: test result information not stored before in any field of this table (maximum 250 characters).

*Tests* table:

- **test_id**: unique test identifier (number between 0 and 9999). It is a mandatory field.
- **test_name**: name of the test, in English (maximum 60 characters).
- **spanish_name**: name of the test in Spanish (maximum 60 characters).
- **speciality**: hospital speciality to which the test corresponds (number between 0 and 99).

*Laboratories* table:

- **lab_id**: unique laboratory identifier (maximum 10 characters). It is a mandatory field.
- **lab_name**: laboratory name (maximum 60 characters).
- **lab_telephone**: telephone number of the laboratory (9 characters).

*Results* table:

- **result_id**: unique result identifier (number between 0 and 99). It is a mandatory field.
- **result**: type of result (maximum 15 characters).

**Clinical Information Module:**

*Clinical information* table:

- **disease_name**: unique disease identifier (links to *diseases* table). It is a mandatory field.
- **SIP**: unique numerical personal identifier of each patient (links to *patients* table). It is a mandatory field.
- **information_date**: when clinical information was collected (date format: YYYY-MM-DD). It is a mandatory field.
- **diagnosis_date**: when the disease was diagnosed (date format: YYYY-MM-DD).
- **centre_number**: identifier number of the hospital or health centre where the patient was attended (links to *health centres* table).
- **start_date**: when the disease began (date format: YYYY-MM-DD).
- **pathological_basis**: information about prior patient’s pathology (maximum 250 characters).
- **immunosupresor_state**: information about immunosupressor state of the patient at the moment this clinical information was collected (maximum 50 characters).
- **vaccinated_state**: information about the vaccination status of the patient (select one among these options: ‘complete’, ‘incomplete’, ‘unknown’). It cannot be a blank space.
- **comments**: Information about clinical information not stored before in any field of this table (maximum 250 characters).

*Risk factors* table:

- **riskfactor_id**: unique risk factor identifier (number between 0 and 9999). It is a mandatory field.
- **riskfactor_name**: name of the risk factor (maximum 60 characters).
- **riskfactor_description**: characteristics and description of the risk factor (maximum 200 characters).

*Protector factors* table:

- **protectorfactor_id**: unique protector factor identifier (number between 0 and 9999). It is a mandatory field.
- **protectorfactor_name**: name of the protector factor (maximum 60 characters).
- **protectorfactor_description**: characteristics and description of the protector factor (maximum 200 characters).

*Vaccines* table:

- **vaccine_name**: name of the vaccine and unique identifier (maximum 50 characters). It is a mandatory field.
- **vaccine_type**: vaccine description (maximum 100 characters).
- **comments**: vaccine information not stored before in any field of this table (maximum 250 characters).

*Vaccinations* table:

- **vaccine_name**: name of the vaccine (links to *vaccines* table). It is a mandatory field.
- **SIP**: unique numerical personal identifier of each patient (links to *patients* table). It is a mandatory field.
- **vaccination_date**: when the vaccine was administered (date format: YYYY-MM-DD).
- **vaccine_dose**: dose administered (maximum 30 characters).
- **comments**: vaccination information not stored before in any field of this table (maximum 250 characters).

*Pathogen test* table:

- **SIP**: unique numerical personal identifier of each patient (links to *patients* table). It is a mandatory field.
- **pathogen_id**: unique pathogen identifier (links to *pathogens* table). It is a mandatory field.
- **last_negative_date**: when last negative test appeared (date format: YYYY-MM-DD).
- **first_positive_date**: when first positive test appeared (date format: YYYY-MM-DD).
- **comments**: pathogen test information not stored before in any field of this table (maximum 250 characters).

*Diseases* table:

- **disease_name**: name and unique disease identifier (maximum 150 characters). It is a mandatory field.

*Symptoms* table:

- **symptom_id**: unique symptom identifier (between between 0 and 9999). It is a mandatory field.
- **symptom_name**: name of the symptom (maximum 60 characters).
- **symptom_description**: characteristics and description of the symptom (maximum 200 characters).

*Signs* table:

- **sign_id**: unique sign identifier (number between 0 and 9999). It is a mandatory field.
- **sign_name**: name of the sign (maximum 60 characters).
- **sign_description**: characteristics and description of the sign (maximum 200 characters).

Note: A sign is a clinical feature of a disease or condition that the doctor looks for. It is an *objective* evidence of disease that can be seen or measured. A symptom is a *subjective* evidence of disease that the patient feels or complains about. Symptoms cannot be directly observed. Both signs and symptoms are assessed in order to make a diagnosis.

**Processes Module**:

*Laboratory processes* table:

- **process_id**: unique process identifier (by default a number between 0 and 4294967295). It is a mandatory field.
- **sample_id**: unique sample identifier (links to samples table).
- **extraction_id**: unique extraction identifier (links to *extractions* table).
- **region_name**: unique region identifier (links to *regions* table).
- **primer_seq**: unique primer identifier (links to *primers* table).
- **amplification_id**: unique amplification identifier (links to *amplifications* table).
- **sequencing_id**: unique sequencing identifier (links to *sequencing* table).
- **typing_id**: unique typing identifier (links to *typing* table).
- **comments**: process information not stored before in any field of this table (maximum 250 characters).

*Extractions* table:

- **extraction_id**: unique extraction identifier (code of 11 digits). It is a mandatory field.
- **extraction_date**: when extraction was performed (date format: YYYY-MM-DD).
- **kit**: kit used in the extraction (maximum 30 characters).
- **comments**: extraction information not stored before in any field of this table (maximum 250 characters).

*Primers* table:

- **primer_seq**: unique primer sequence identifier (sequence code, maximum 250 characters). It is a mandatory field.
- **primer_name**: name of the primer (maximum 30 characters).
- **position**: number of nucleotide position of the primer in the sequence (number between 0 and 4294967295).
- **sense**: sense of the primer (select one of this options: ‘sense’, ‘antisense’, ‘unknown’). It cannot be a blank space.
- **comments**: primer information not stored before in any field of this table (maximum 250 characters).

*Regions* table:

- **region_name**: unique genome region identifier (maximum 15 characters). It is a mandatory field.
- **comments**: region information not stored before in any field of this table (maximum 250 characters).

*Amplifications* table:

- **amplification_id**: unique amplification identifier (code of 11 digits). It is a mandatory field.
- **amplification_result**: result of amplification (select one of these options: ‘+’, ‘-’, ‘unknown’). It could not be a blank space.
- **comments**: amplification information not stored before in any field of this table (maximum 250 characters).

*Sequencing* table:

- **sequencing_id**: unique sequencing identifier (code of 11 digits). It is a mandatory field.
- **sequencing_result**: result of the sequencing (select one of these options: ‘+’, ‘-’, ‘unknown’). It cannot be a blank space.
- **date_sent**: when the sample was sent for sequencing (date format: YYYY-MM-DD).
- **date_arrived**: when sequencing arrived (date format: YYYY-MM-DD).
- **comments**: sequencing information not stored before in any field of this table (maximum 250 characters).

*Typing* table:

- **typing_id**: unique typing identifier (code of 11 digits). It is a mandatory field.
- **typing_result**: result of typing (maximum 25 characters).
- **comments**: typing information not stored before in any field of this table (maximum 250 characters).

**Bibliography Module:**

*Researchers* table:

- **researcher_id**: unique researcher identifier (code of 7 characters). It is a mandatory field.
- **group_id**: unique research groups identifier (links to *research groups* table).
- **first_name**: name of the researcher (maximum 20 characters).
- **last_name1**: first last name of the researcher (maximum 100 characters).
- **last_name2**: seconde last name of the researcher (maximum 100 characters).
- **DNI**: national identity card of the researcher (9 characters). This is a unique identifier for each researcher.
- **address_street**: street name (maximum 150 characters).
- **address_postalcode**: postal code (5 characters).
- **address_place**: name of the place (maximum 100 characters).
- **telephone**: telephone number (9 characters).
- **e-mail**: e-mail address (maximum 40 characters).
- **comments**: researcher information not stored before in any field of this table (maximum 250 characters).

*Research groups* table:

- **group_id**: unique research group identifier (code of 10 characters). It is a mandatory field.
- **group_name**: name of the research group (maximum 200 characters). This is a unique identifier for each research group.
- **group_description**: other characteristics of the research group (maximum 250 characters).
- **comments**: research group information not stored before in any field of this table (maximum 250 characters).

*Bibliographic references* table:

- **reference_id**: unique reference identifier (by default a number between 0 and 4294967295). It is a mandatory field.
- **title**: title of the reference (maximum 250 characters). There cannot be two identical titles.
- **publication_date**: when the reference was published (date format: YYYY-MM-DD).
- **publication**: name of the journal (maximum 100 characters).
- **acceptance_date**: when the reference was accepted by the journal (date format: YYYY-MM-DD).
- **link**: where the paper is located (maximum 100 characters).
- **comments**: bibliographic reference information not stored before in any field of this table (maximum 250 characters).

*Authors* table:

- **author_id**: unique author identifier (code of 6 characters). It is a mandatory field.
- **author_firstname:** name of the author (maximum 50 characters).
- **author_lastname1**: first last name (maximum 100 characters).
- **author_lastname2**: second last name, if exists (maximum 100 characters).
- **comments**: author information not stored before in any field of this table (maximum 250 characters).

**Results Module:**

*Alignments* table:

- **alignment_name**: unique alignment identifier, name of the file (maximum 40 characters). It is a mandatory field.
- **date_aobtained**: when the alignment was obtained (date format: YYYY-MM-DD).
- **aarchive_location**: where the alginment is (maximum 50 characters).
- **alignment_type**: type of alignment (select one of these options: ‘multiple alignment’, ‘database search’, ‘pairwise comparison’, ‘unknown’). It can be a blank space.
- **program**: program used to obtain the alignment (maximum 30 characters).
- **score**: score value of alignment (maximum 10 digits).
- **p-value**: p-value of alignment (a decimal number of 10 digits with 6 decimals).
- **e-value**: e-value of alignment (a decimal number of 10 digits with 6 decimals).
- **comments**: alignment information not stored before in any field of this table (maximum 250 characters).

*Phylogenetic trees* table:

- **tree_name**: unique phylogenetic tree identifier, name of the file (maximum 40 characters). It is a mandatory field.
- **alignment_name**: unique alignment identifier (links to *alignments* table).
- **date_tobtained**: when the tree was obtained (date format: YYYY-MM-DD).
- **tarchive_location**: where the tree is (maximum 50 characters).
- **model**: model used to obtain the tree (select one of this options: ‘JC’, ‘F81’, ‘K80’, ‘HKY’, ‘TNef’, ‘TN’, ‘K81’, ‘K81uf’, ‘TIMef’, ‘TIM’, ‘TVMef’, ‘TVM’, ‘SYM’, ‘GTR’, ‘unknown’). It can be a blank space.

| JC | Jukes and Cantor (Jukes and Cantor, 1969) |
| --- | --- |
| F81 | Felsenstein 81 (Felsenstein, 1981) |
| K80 | Kimura 80 (=K2P) (Kimura, 1980) |
| HKY | Hasegawa, Kishino, Yano 85 (Hasegawa, Kishino, Yano, 1985) |
| TNef | Tamura-Nei equal frequencies |
| TN | Tamura-Nei (Tamura and Nei, 1993) |
| K81 | Two transversion parameters model 1 (=K81 =K3P) (Kimura, 1981) |
| K81uf | Two transversion parameters model 1 unequal frequencies |
| TIMef | Transitional model equal frequencies |
| TIM | Transitional model |
| TVMef | Tranversional model equal frequencies |
| TVM | Tranversional model |
| SYM | Symmetrical model (Zharkihk, 1994) |
| GTR | General time reversible (=REV) (Tavaré, 1986) |

- **program**: program used to obtain the tree (maximum 20 characters).
- **options_invariants**: information about the invariants used (decimal number of 5 digits with 4 decimals).
- **options_gamma**: gamma distribution shape parameter (alpha, decimal number of 5 digits with 4 decimals). Distribution to correct the heterogenity of evolutionary tases per site.
- **options_comments**: options information not stored before in any field of this table (maximum 250 characters).
- **lrt_value**: test value of likelihood rate (decimal number of 15 digits with 4 decimals).
- **lrt_pvalue**: p-value of LRT (decimal number of 8 digits with 6 decimals).
- **lrt_df**: degrees of freedom of LRT (maximum 2 digits).
- **aic**: AIC value of the tree (decimal number of 15 digits with 4 decimals).
- **comments**: phylogenetic tree information not stored before in any field of this table (maximum 250 characters).
